# Complete androgen insensitivity syndrome presenting with bilateral adnexal masses and mixed gonadal histopathology

**DOI:** 10.1007/s00404-026-08402-6

**Published:** 2026-04-09

**Authors:** Ayse Gizem Yildiz, Ahmet Kurt, Ilayda Deniz Cengiz, Sehbal Arslankoz, Ismail Burak Gultekin

**Affiliations:** 1 Department of Obstetrics and Gynecology, Ankara Etlik City Hospital, 06010 Ankara, Turkey; 2 Department of Pathology, Ankara Etlik City Hospital, Ankara, Turkey

**Keywords:** Androgen insensitivity syndrome, Prophylactic gonadectomy, Sertoli cell adenoma, Leydig cell tumor

## Abstract

**Purpose:**

Complete androgen insensitivity syndrome (CAIS) is a rare X-linked recessive disorder due to androgenreceptor mutations, characterised by a 46,XY karyotype, female phenotype, and undescended testes. This reportaims to illustrate the clinical management and the rare synchronous pathology of multiple gonadal tumors in a 60-year-old phenotypic female with long-standing CAIS.

**Methods:**

A 60-year-old patient presented following the incidental detection of bilateral adnexal masses. Diagnosticevaluation included imaging (USG/MRI) to assess internal reproductive organs and gonadal morphology, alongsidehormonal analysis (LH, testosterone, and estradiol). A laparoscopic bilateral gonadectomy was performed to managethe suspected neoplasia.

**Results:**

Imaging demonstrated the absence of the uterus and ovaries, confi rming bilateral solid gonadal lesions.Hormonal analysis showed elevated LH with normal testosterone and estradiol levels. Histopathology of the excisedtissues revealed a complex and heterogeneous presentation: the left gonad contained a Sertoli cell tumor, a Leydigcell tumor, and sclerotic seminiferous tubules; the right gonad showed a Sertoli cell adenoma.

**Conclusion:**

This rare synchronous pathology illustrates the signifi cant heterogeneity of neoplasia associated withCAIS. Current evidence supports individualized postpubertal gonadectomy to balance the risk of malignancy againstthe benefi ts of endogenous hormonal production. The fi ndings emphasize that long-term follow-up and tailoredsurgical timing are essential components in the clinical management of CAIS.

## What does this study adds to the clinical work

**Table:** 

Even in advanced age, CAIS can present with previously unrecognized gonads harboring multiple distinct tumor types. This case emphasizes that tumor risk is not only persistant but also histologically diverse, requiring individualized clinical management.	

## Introduction

Testicular feminization syndrome, also referred to as complete androgen insensitivity syndrome (CAIS), is a rare X-linked (locus Xq11–12) recessive disorder characterized by complete resistance to androgen action in individuals with a 46,XY karyotype. First described by John Morris in 1953 [1], CAIS has an estimated prevalence of approximately 1 in 20,000 live births and, in one study, was identified as the third most common cause of primary amenorrhea [2]. Although these individuals possess a male chromosomal and gonadal sex, they typically present as phenotypically, anatomically, and socially female, with normal breast development but sparse or absent pubic and axillary hair at puberty [3, 4].

Androgen insensitivity syndrome (AIS) includes complete (CAIS), partial (PAIS), and mild forms (MAIS), depending on the severity of androgen receptor (AR) dysfunction. Within PAIS, the severity of androgen resistance is further stratified into five grades [5]:

- *Grade 1:* Typical female external genitalia with the development of androgen-dependent pubic and axillary hair at puberty.
- *Grade 2:* Female phenotype with moderate clitoromegaly and partial fusion of the posterior labia.
- *Grade 3:* Ambiguous genitalia characterized by a phallic structure intermediate between a clitoris and penis, a perineal urethral opening, and labioscrotal folds within a urogenital sinus.
- *Grade 4:* Predominantly male phenotype with perineal hypospadias, bifid scrotum, cryptorchidism, and a small penis.
- *Grade 5:* Mild undermasculinization presenting as isolated hypospadias or micropenis.

This spectrum of clinical presentation is primarily caused by mutations in the AR gene, often located within its ligand-binding domain (LBD) or DNA-binding domain (DBD), impairing androgen binding, DNA interaction, or downstream gene transcription [6]. These mutations result in variable degrees of genital development. Interestingly, the same AR mutation can lead to different phenotypic expressions even within the same family, emphasizing the complexity of genotype–phenotype correlation [4]. Additionally, a partial defect in the AR gene in males may present with a spectrum ranging from undervirilization to infertility or gynecomastia [7].

Internally, individuals with CAIS lack Müllerian-derived structures, such as the uterus, fallopian tubes, and upper vagina. Despite normal intrauterine testosterone production, the unresponsiveness of target tissues to androgens prevents masculinization of male genitalia and secondary sexual characteristics [8]. Instead, they exhibit a short, blind-ending vaginal pouch, and the testes, though developed, typically remain undescended—located most commonly in the inguinal canal (60%), abdomen (21%), or occasionally within the labia majora [9].

Gross examination of the gonads often reveals testes that are slightly smaller than normal, sometimes with cystic changes, fibromuscular tissue, and well-demarcated cream-colored nodules [10]. This abnormal testicular development is associated with an increased risk of germ cell neoplasia, particularly in cryptorchidism, with the risk increasing with age. Although Sertoli-Leydig cell tumors are rare in CAIS, they are typically benign [11, 12]. However, some studies suggest that the risk of malignancy may increase in the post-pubertal period due to the potential malignant transformation of the gonads [13].

Current guidelines do not recommend routine early prophylactic gonadectomy. However, due to the progressive risk of tumor development over time, post-pubertal gonadectomy on an individualized basis under close follow-up is advised [14–16].

This case report describes the diagnostic approach and clinical management of a phenotypic female with a 46,XY karyotype, in whom bilateral adnexal masses were incidentally identified during routine follow-up.

## Case report

In this case, a 60-year-old married phenotypic female presented to the gynecology outpatient clinic in December 2023 with MRI results obtained externally during routine follow-up. The patient had been diagnosed with CAIS during adolescence as part of the evaluation for primary amenorrhea. Chromosomal analysis had previously revealed a 46,XY karyotype. However, she reported no chronic medical conditions and no sexual dysfunction. Her surgical history included only inguinal hernia repair, with no other interventions.

On physical examination, she exhibited normal female external genitalia, well-developed breasts, and absence of axillary and pubic hair—a clinical pattern consistent with CAIS. No uterus or ovaries were visualized on ultrasound; instead, bilateral solid adnexal masses were identified, raising suspicion for undescended testicular tissue. Pelvic MRI revealed a well-encapsulated lesion in the left adnexal region, measuring approximately 45 × 35 mm at its largest dimension. The lesion appears heterogeneously hyperintense on T2-weighted sequences and mildly hypointense on T1-weighted sequences.

Laboratory tests showed a hemoglobin level of 13.3 g/dL and a white blood cell count of 5000/mm^3^. Tumor markers were within normal ranges. Hormonal evaluation revealed elevated luteinizing hormone (LH) levels and testosterone within the normal range, consistent with androgen resistance. Estradiol levels were within normal limits. Due to the suspected presence of intraabdominal testicular tissue and the potential risk of malignancy, diagnostic laparoscopy was planned.

**Fig. 1:**
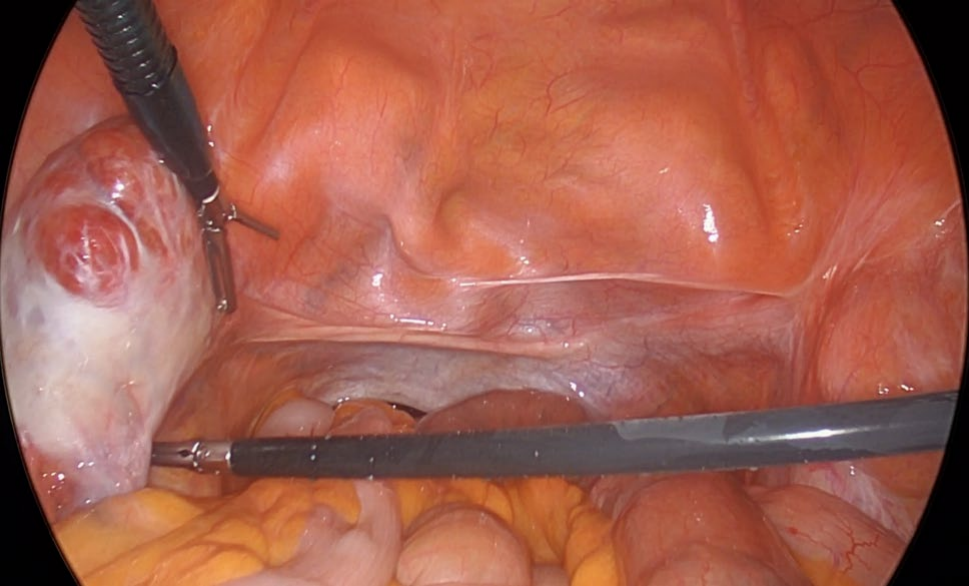
Laparoscopic view demonstrating absence of uterus (uterine agenesis) and appearance of left gonad.

**Fig. 2:**
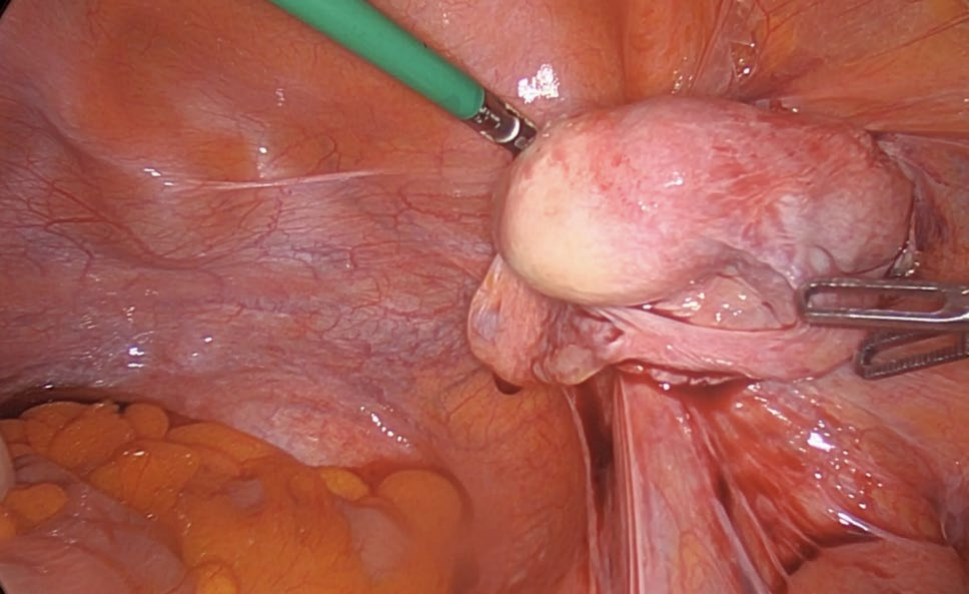
Laparoscopic view of the right gonad showing macroscopic testicular features.

Intraoperatively, a solid lesion approximately 4 cm in the left adnexal region and another approximately 3 cm in the right were observed. Both appeared macroscopically consistent with testicular tissue (Figs. 1, 2). Frozen section analysis confirmed benign testicular tissue in both masses.

**Fig. 3:**
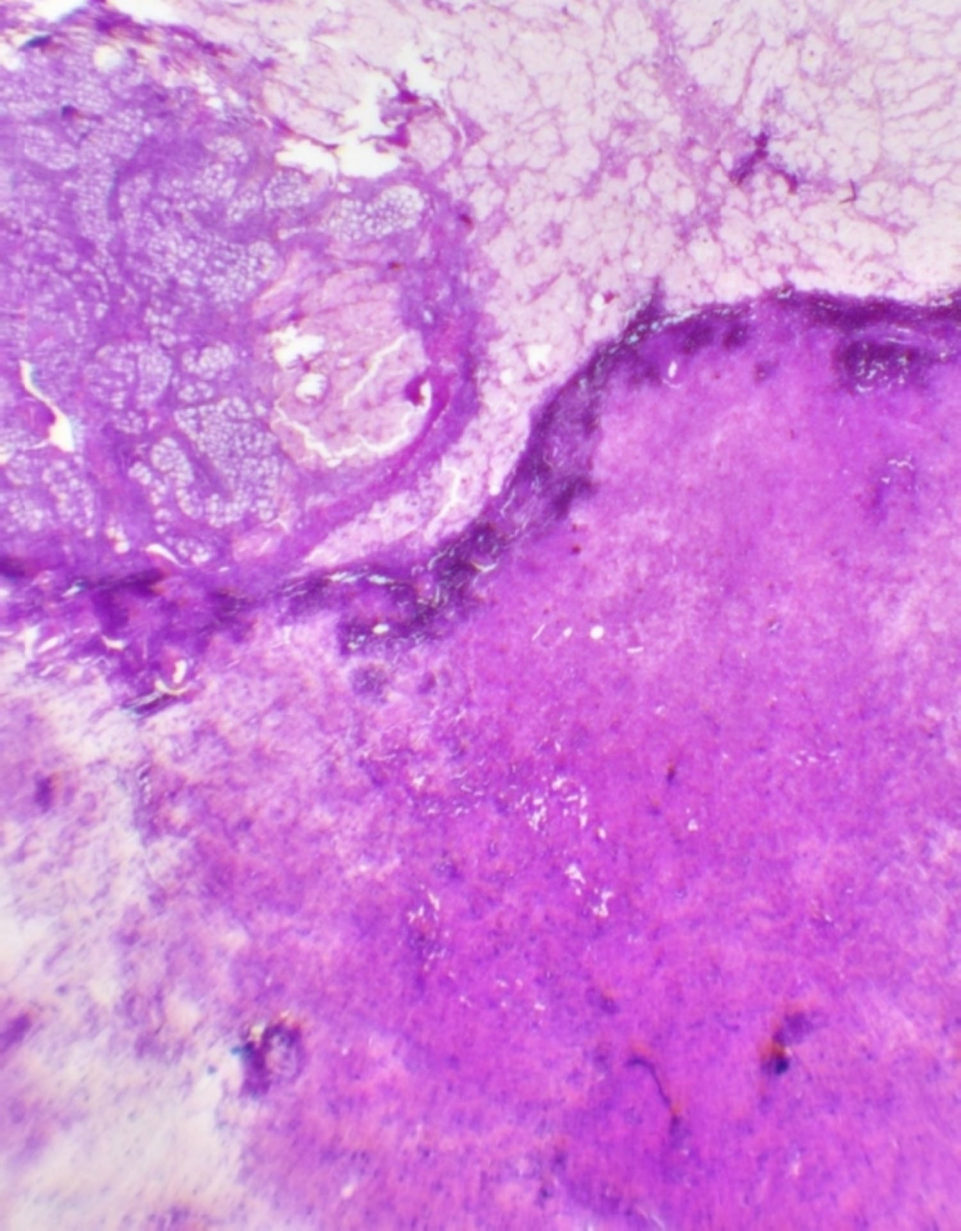
Composite Histopathological Features of the Left Adnexal Mass

**Fig. 4:**
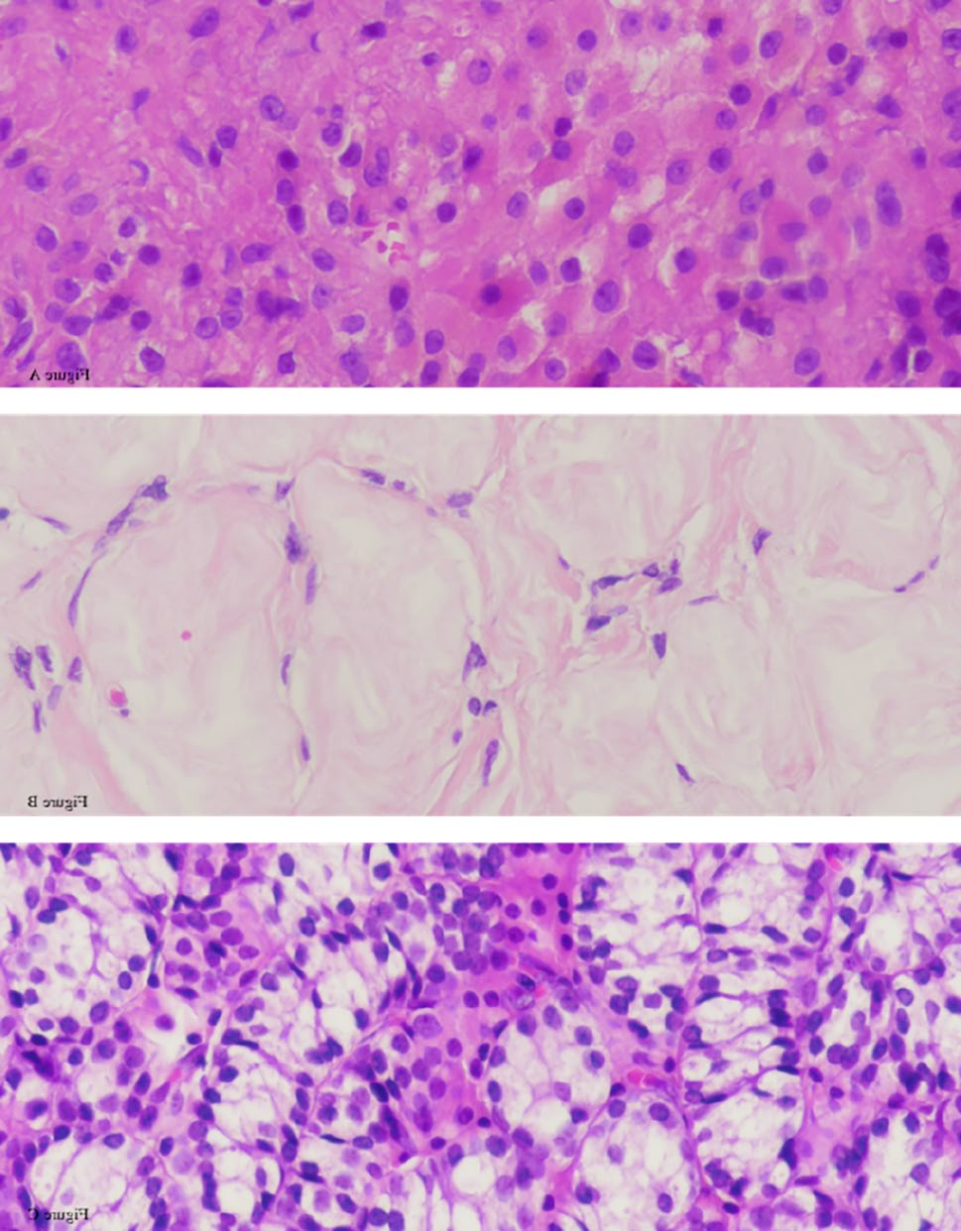
Histopathological Features Observed in the Left Gonad. **A** Leydig cell tumor component. Tumor cells are polygonal with abundant densely eosinophilic cytoplasm, round nuclei and central nucleoli. **B** Sclerotic seminiferous tubules. **C** Sertoli cell adenoma component composed of closely packed tubules which are lined with immature Sertoli cells.

**Fig. 5:**
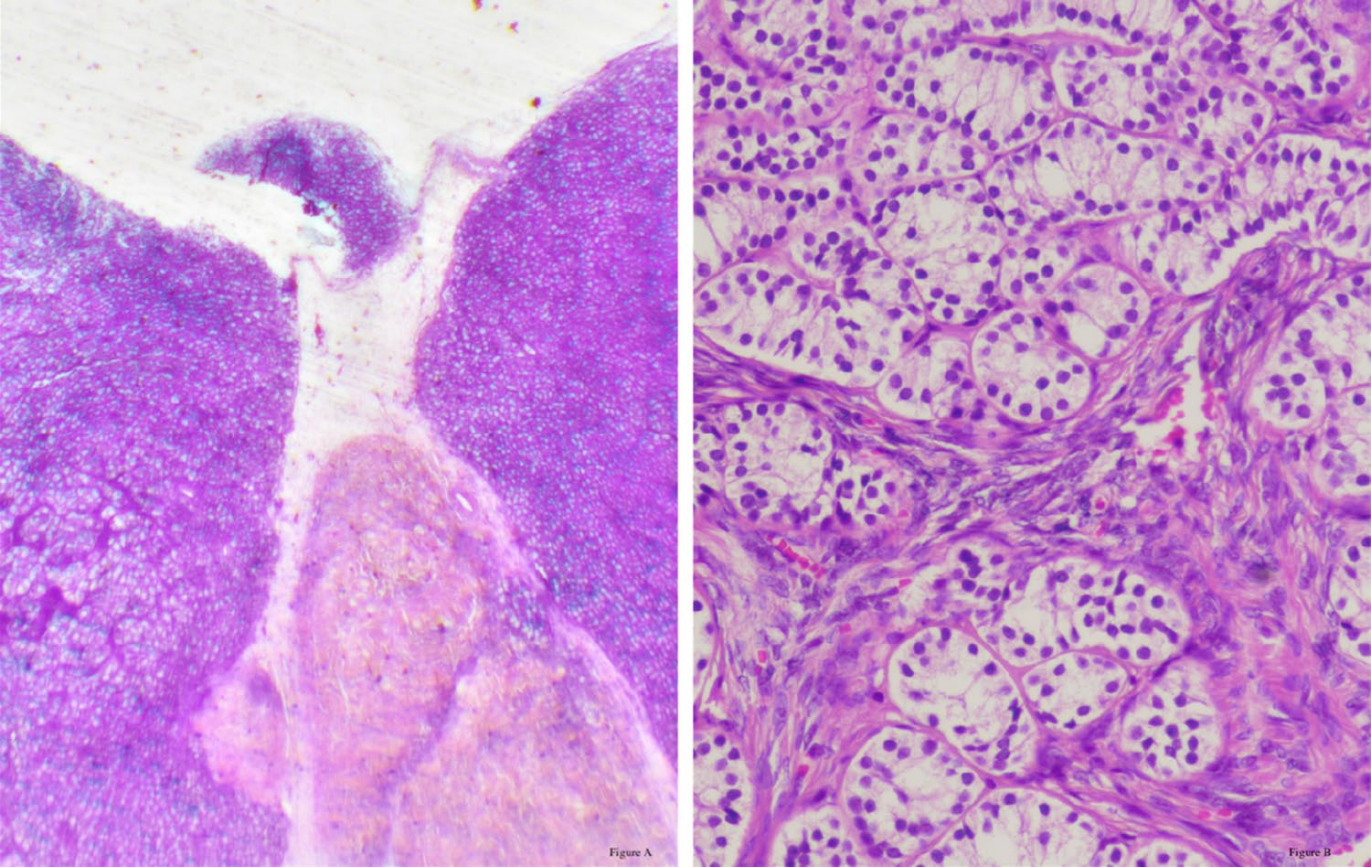
Histopathological Features of the Right Adnexal Mass: Sertoli Cell Adenoma. **A** Right adnexal mass lesion at low magnification. Sertoli cell adenoma areas with smooth borders are localized on the right and left sides of the figure. **B** Back-to-back tubule structures lined with immature Sertoli cells.

Histopathological evaluation revealed distinct but complementary findings in both gonads. In the left gonad, a composite pattern was observed, consisting of a Sertoli cell tumor with smooth, well-circumscribed borders composed of tightly packed tubules lined by immature Sertoli cells; sclerotic seminiferous tubules exhibiting fibrotic changes and architectural disorganization; and a well-demarcated Leydig cell tumor composed of polygonal cells with abundant eosinophilic cytoplasm and central nucleoli. In contrast, the right gonad demonstrated more uniform pathology, predominantly composed of Sertoli cell adenoma areas located bilaterally within the tissue section, showing closely packed back-to-back tubule formations lined by immature Sertoli cells (Figs. 3, 4, and 5).

Left adnexal mass lesion at low magnification. A Sertoli cell tumor is observed on the upper left side, with smooth borders. There are sclerotic seminiferous tubule structures in the upper right corner. A well-circumscribed Leydig cell tumor area is seen on the lower right.

Sertoli cell tumors are rare in patients with disorders of sex development and are typically benign, although malignancy has been reported in a minority of cases. The coexistence of Sertoli cell adenoma and Leydig cell tumor within bilateral gonadal structures in a patient with CAIS is a rare and noteworthy presentation. Sclerotic seminiferous tubules reflect functionally impaired testicular tissue, typically lacking spermatogenesis.

Current recommendations advise against early prophylactic gonadectomy; however, due to the progressive risk of neoplasia with age, individualized post-pubertal gonadectomy following careful surveillance is considered appropriate.

This case highlights a rare disorder of sex development characterized by synchronous Sertoli and Leydig cell proliferative changes in a 46,XY phenotypic female. Long-term follow-up is essential to monitor for both malignant transformation potential.

## Discussion

AIS, formerly known as testicular feminization syndrome, is a rare X-linked recessive disorder characterized by a 46,XY karyotype and resistance to androgen action due to mutations in the AR gene located on Xq11–12. Despite having male chromosomal and gonadal sex, affected individuals present with female external genitalia, absent Müllerian structures, well-developed breasts at puberty, sparse or absent pubic and axillary hair, and a short, blind-ending vagina. The condition typically becomes clinically apparent during evaluation for primary amenorrhea or bilateral inguinal hernia in infancy or adolescence [10]. Most cases are diagnosed in adolescence due to lack of menstruation despite normal secondary sexual characteristics.

These individuals are typically raised as females and socially identify as such. The phenotypic variability observed in AIS has been explored in large cohorts. In a comprehensive analysis of 278 cases, Ahmed et al. found a wide range of AR mutations leading to variable AIS phenotypes, with limited genotype–phenotype correlation [17].

Patients with CAIS carry a lifelong risk of gonadal malignancy due to undescended testes [18]. While this risk remains relatively low during the prepubertal period, it increases with age. Therefore, close histopathological assessment following gonadectomy is essential. A large case series reported germ cell neoplasia in situ (GCNIS) and malignancy predominantly in post-pubertal individuals, supporting current recommendations for delayed and individualized gonadectomy under careful clinical surveillance [19]. According to Fagouri et al., the lifetime risk of gonadal tumors in CAIS ranges from 5 to 10%, reaching up to 33% after the age of 50 [12].

The presence of these diverse lesions, including testicular tumors and dysgenetic tubules, underscores the broad histopathological spectrum seen in patients with CAIS and highlights the importance of thorough gonadal examination in such cases. Although these tumors are often benign, malignant transformation has been reported in the literature. Therefore, long-term follow-up and timely surgical excision remain essential to mitigate potential oncologic risk, especially in post-pubertal or older individuals.

This synchronous presentation is rare, but has been previously described and highlights the importance of timely gonadectomy after puberty [20]. In rare case reports, have demonstrated that there is the presence of synchronous Leydig cell hyperplasia and atrophic seminiferous tubules even at earlier ages [21].

Current guidelines advise against early prophylactic gonadectomy to allow spontaneous puberty and full breast development. However, given the progressive tumor risk with age, individualized post-pubertal gonadectomy is considered the most appropriate approach. In this patient, bilateral gonadectomy facilitated a definitive diagnosis and effectively removed the potential risk of malignancy. This case is notable for its delayed diagnosis and the synchronous benign proliferative lesions. It highlights the importance of recognizing CAIS in atypical clinical presentations and underscores the need for multidisciplinary, long-term management.

## Conclusion

Although the diagnosis was initially made during puberty because of amenorrhea, long-term follow-up was not established, leading to delayed surgical management. The incidental detection of bilateral adnexal masses later in life allowed for definitive histopathological confirmation. This case emphasizes the importance of lifelong monitoring in CAIS patients and the role of individualized surgical timing to mitigate the risk of gonadal malignancies.

## Data Availability

No datasets were generated or analyzed during the current study.
